# Predictive validity of a parental questionnaire for identifying children with developmental language disorders

**DOI:** 10.3389/fpsyg.2023.1110449

**Published:** 2023-06-19

**Authors:** Alejandra Auza B., Chiharu Murata, Christian Peñaloza

**Affiliations:** ^1^Language and Cognition Laboratory, Hospital General Dr. Manuel Gea González, Mexico City, Mexico; ^2^Departamento de Metodología de la Investigación, Instituto Nacional de Pediatría, Mexico City, Mexico; ^3^Departamento de Fonoaudiología, Facultad de Medicina, Universidad de Chile, Santiago, Chile

**Keywords:** parental questionnaires, developmental language disorder (DLD), early identification, Spanish-speaking children, parental linguistic concerns

## Abstract

**Background:**

The underdiagnosis of developmental language disorder (DLD) in children is a serious problem in developing countries with limited resources. It has long been noted that the concerns parents have about their children’s health and development are richly informative, and if this information can be used for diagnosis, it may provide a means to address the problem of underdiagnosis of DLD. This study aimed to quantify the utility of parental linguistic concern questions (PLCQ) on the identification of language disorders in monolingual Spanish-speaking children in Mexico. It also explored whether a combination of biological and environmental conditions questions (BECQ) might improve the performance of a screening test to identify DLD.

**Methods:**

A total of 680 monolingual Mexican Spanish-speaking children and their parents from urban areas in Mexico participated in the study. The distribution of responses to questions about DLD concerns was compared between 185 children diagnosed with DLD and 495 control subjects, and multiple logistic regression analysis was performed to select questions with high predictivity, based on the Akaike information criterion. The diagnostic utility of the questions was assessed by receiver operating characteristic (ROC) curves, stratum-specific likelihood ratios (SSLRs), and changes in pretest and post-test probabilities of DLD. A similar procedure was used to explore whether adding BECQ would improve the diagnostic utility of questions about DLD concerns using data of 128 children.

**Results:**

Four questions regarding parental linguistic concerns were found to be useful in identifying children with DLD. When all four concerns were present, the SSLR was 8.79, while it was only 0.27 when there were no concerns at all. The estimates of DLD probability increased from 0.12 to 0.55 at pretest and post-test. On the other hand, the BECQ did not perform as well as the PLCQ in identifying DLD, and the improvement in diagnostic performance it provided was limited to one question.

**Conclusion:**

The parental questionnaire can be used as a screening tool to help in identifying children with DLD. The data presented in this study underscore the importance of considering linguistic parental concerns as part of the screening process. This is a realistic option to provide a solution to the current problem of underdiagnosis of DLD in Mexico.

## 1. Introduction

Parental questionnaires are tools that provide access to information about children’s language and communication, based on the daily experiences of parents and sometimes teachers of young children. They have emerged as an alternative to the lack of standardized assessment tools in certain sociocultural contexts, such as migrant populations in countries where there are no established reference standards for language development (Restrepo, 1998). Screening for developmental language disorder (DLD) among children in speech and language clinics is a challenge when standardized tests for the monolingual or bilingual population are not available, and even more when there is a lack of knowledge on children’s performance in the home language (Abutbul-Oz and Armon-Lotem, 2022).

Parental questionnaires have been used as a supplement to formal assessments since they provide a way to describe children’s language skills (Restrepo, 1998). Parental questionnaires also enable professionals to understand the expectations that adults have regarding their children’s skills, as well as their overall knowledge about child development. Additionally, they can help parents become more involved in their children’s language development (Thal et al., 1999; Bishop and McDonald, 2009; Guiberson et al., 2011). However, this consideration must be examined within the context of Latin American families. While middle-class English speakers may report positive involvement in their children’s school and therapeutic activities, it’s important to understand how this may differ from the experiences of Latin American families. Moreover, Latin American parents often report not having the same attitude, particularly because they perceive it as an intrusion into the work of professionals and a challenge to their own authority (Rodriguez and Olswang, 2003).

In general, two types of PQ can be identified. The first type focuses on evaluating language development. In these assessments, parents are asked to report on the emergence of a set of communicative and linguistic resources in their children. The MacArthur-Bates Communicative Development Inventory (MB-CDI) (Fenson et al., 1993), Language Developmental Survey (LDS), and the Children’s Communication Checklist-2 (CCC-2) (Bishop, 2003) belong to this category. Each one of them has adaptations for Spanish speakers and its use has increased as screening to identify children with language delays or difficulties at an early age. For example, Bishop and McDonald (2009) observed that the combined administration of the CCC-2 with language tests achieved a good degree of specificity, but not of sensitivity when discriminating between children with and without language disorders. However, the use of the CCC-2 always improved the results compared to the discriminating power of language tests alone.

The second type of questionnaire focuses on parental concerns regarding language development, and also collects information from the child’s environment. An example is the PQ proposed by Restrepo (1998). She constructed this questionnaire to aid in the assessment of Spanish-speaking children, and when combined with the mean length of utterances (MLU) and the number of grammatical errors per clause, it can provide valuable information. Her questionnaire includes questions related to language milestones, biological, social, and linguistic concerns, which are answered with yes or no. However, there is no information available regarding the selection of the questions or whether the severity of reported difficulties is considered when parents respond to multiple questions.

The use of parental reports is based on the idea that they are equally sensitive to the formal assessment of a professional in the field to evaluate children’s communicative and linguistic abilities in different cultural and linguistic contexts (Guiberson and Rodríguez, 2010). However, some authors have pointed out a discrepancy between children who qualify as having language difficulties based on low scores in standardized tests and those who are reported as having difficulties by their parents. Law et al. (2011) found that only a small percentage of children identified as having language difficulties through a series of standardized tests had been detected by teachers in a school setting and referred to language services. Tomblin et al. (1997) observed a similar pattern, noting that only 29% of children identified as having language impairment through formal testing had been previously identified by their parents or school services. This percentage increased only to 39% in the case of children with severe difficulties. Finally, in the study by Bishop and McDonald (2009), more than half of the children who obtained low scores on language tests had not been detected by their parents and teachers. An explanation for this discrepancy may lie in the fact that parental reports are completed without the assistance of a professional. This means that clinicians do not directly interview the parents, but instead parents complete the questionnaires themselves. However, other factors may be contributing to this discrepancy. For instance, parents may be more attentive to certain linguistic domains, such as speech-sound disorders, that are not evaluated in the tests. Additionally, parents and teachers may not be aware of difficulties in specific areas of language that are typically present between the ages of 4 and 16 (Caraveo-Anduaga et al., 2002). Therefore, more severe language problems, such as difficulties in understanding or producing grammar, may not be noticeable to parents or may be masked by other academic or social interaction issues. This raises the question of how parents interpret the questions on the questionnaires and compare them to what they observe in their child’s daily life (Bishop and McDonald, 2009). The classification and terminology used to describe developmental language disorder (DLD), previously referred to as specific language impairment (SLI), can be confusing for professionals and families of children with this condition due to the varying degrees of severity and persistent difficulties in structural language, which can result in functional, social, and educational problems (Serra, 2022).

On the other hand, many undetected children belong to socially vulnerable groups, which may indicate that tests should be sensitive to the social environment, or even certain cultural expectations about children, that could be influencing parents’ judgment (Keegstra et al., 2007; Bishop, 2014).

When clear questions are provided, parents can be valuable resources for identifying children with language difficulties or supplementing formal assessments. However, the use of questionnaires, like any assessment tool, must take into account the social and cultural context of the population for which they are intended. The commonly considered predictive values are sensitivity, specificity, negative predictive value (NPV), and positive predictive value (PPV). The area under the curve (AUC), resulting from receiver operating characteristic (ROC) analysis, provides an estimate of the screening or diagnostic test’s discriminative power. A recent systematic review analyzed screening tools for language, showing that the predictive validity data from all sample studies demonstrated a mean sensitivity of 77.7% and a PPV of 66.56%. This result indicates that screening tools for language are more effective and even achieve higher sensitivity, specificity, and negative predictive value than direct child assessment for language development (Sim et al., 2019).

Recently, a study was conducted to determine the effectiveness of a two-step procedure for identifying language difficulties in monolingual Mexican Spanish-speaking children (Auza et al., 2023). This procedure combined a grammatical screener with a short parental questionnaire (PQ). The results showed that both the grammatical screener and the PQ were effective in identifying children with developmental language disorder (DLD) between the ages of 4;0 and 6;11 years old. This was indicated by the stratum-specific likelihood ratios (SSLR) of the PQ, as well as the positive and negative likelihood ratios (LR+ and LR−) of a screening test called the “Tamiz de Problemas de Lenguaje” (TPL) (Auza et al., 2018). However, it is important to note that in this study, only eight linguistic concern questions were included in the questionnaire (see Supplementary Appendix 1, section I). The post-test probability for detecting children with DLD between the ages of 4;0 and 4;11 and between 5;0 and 5;11 was found to be 57% before administering the grammatical screener, and for children between 6;0 and 6;11, the post-test probability was 68%.

Another recent research sought to identify a set of factors associated with Spanish-speaking children with DLD (Peñaloza, 2018). To achieve this goal, 36 variables related to medical history, language development, and environmental factors were explored. These variables were selected based on a review of 60 articles on language development in Spanish and English. A questionnaire was constructed using these variables and piloted with 60 families in Mexico City and Querétaro, Mexico. In Peñaloza’s (2018) study, the researchers investigated the association between environmental variables and the detection of DLD. They found that only eight variables differed statistically between children with and without DLD: these variables were: (1) the sex of the child, (2) the occurrence of motor and/or psychological difficulties during the first years of life, (3) the age of producing the first words, (4) the amount of time the child attended preschool, (5) the years of maternal education, (6) the years of paternal education, (7) the presence of a family history of speech or language problems, and (8) time children attended preschool, and years of maternal education (Auza et al., 2019).

Previous literature suggests that both biological and environmental factors may increase a child’s risk of developing language difficulties. For instance, a child’s risk may be affected by the quality of social and communicative interactions between the child and their parents or caregivers (Raviv et al., 2004; Bradley and Corwyn, 2005), family socio-educational level (Pelchat et al., 2003; Pan et al., 2004; Bornstein et al., 2007; Cabrera et al., 2007; Farkas et al., 2015), perinatal conditions, birth weight, premature delivery, parental education, environmental factors, sex of the children, and family history with DLD (Stromswold, 2001; Viding et al., 2003; Newbury et al., 2005, 2009; Chaimay et al., 2006; Bishop et al., 2012; Nudel and Newbury, 2013; Moriano-Gutiérrez et al., 2017) and the age the first words were produced (Prathanee et al., 2007). Therefore, based on an instrument that includes both questions about parental language concerns and biological and environmental conditions of the family and home, the primary objective of this study is to answer the following questions: 1. Do the eight questions in the parental linguistic concern questionnaire (PLCQ) help identify children with DLD? 2. Is it possible to further reduce the eight questions in the PLCQ? 3. If so, what is the diagnostic performance of the reduced PLCQ in screening children with DLD? Additionally, we attempted to answer an additional question: 4. Can a combination of biological and environmental questions improve the diagnostic accuracy of the PLCQ in identifying children with DLD? Our hypothesis is that the questionnaire can provide sufficient information and serve as a screening tool to help identify children with DLD. We predict that the parental concern questions, along with some biological and environmental questions, will improve the performance of the questionnaire.

## 2. Materials and methods

### 2.1. Participants

The study recruited a convenience sample of 680 monolingual Mexican Spanish-speaking children, with 240 4-year-olds, 225 5-year-olds, and 215 6-year-olds, from urban and suburban areas in four different locations in Mexico. The recruitment process involved contacting parents through schools and public health centers and inviting them to participate in the study, regardless of whether they were concerned about their child’s language development. Parents were asked to sign an informed consent form, and children between 6;0 and 6;11 provided verbal assent to participate. The study was approved by the ethics and research committee of the Hospital General Dr. Manuel Gea González, Mexico City. The parents were also asked to complete the parental linguistic concern questionnaire (PLCQ).

### 2.2. Procedure for diagnosis of DLD

Each child underwent three individual evaluation sessions, each lasting approximately 20 min. During the first evaluation, an auditory screening was conducted, and subtests of the Kaufman Assessment Battery for Children 2 (KABC-II), a cognitive test, were administered (Kaufman and Kaufman, 2004), to rule out hearing or cognitive problems. During the second and third sessions, the linguistic tests and language sample were administered, including the morphosyntax subtest of the bilingual English-Spanish language test (BESA) (Peña et al., 2014); the grammatical subtests of the Spanish Clinical Evaluation of Language Fundamentals−Fourth Edition, Spanish (CELF-4) (Semel et al., 2006); the TPL screening test that identifies grammatical difficulties in Mexican Spanish-speaking children between four and six years of age with grammatical problems (Auza et al., 2018); and a language sample, from a retelling of one of the frog stories (Mayer, 1973, 1974; Mayer and Mayer, 1975), for obtaining the percentage of grammatical errors (percentage of ungrammaticality -PU-) per clause (Restrepo, 1998). According to their manuals, these tests demonstrate very good diagnostic accuracy: the BESA is reported to have 87.5% sensitivity and 100% specificity, and CELF-4 Spanish, has 96% sensitivity and 87% specificity. The TPL is reported to have a sensitivity and specificity of 90 and 83% for 4-year-olds, 90 and 84% for 5-year-olds, and of 94 and 92% for 6-year-olds. Children with typical language development (TLD) met the following criteria: (a) 4-year-old children scored above the cut score of 50 on the morphosyntax subtest of BESA; 5- and 6-year-old children scored within one standard deviation from the mean or above on grammatical subtests of the CELF-4 Spanish because it has higher specificity than BESA for this age range; (b) scored above or on the 16th percentile on the TPL screening test; (c) the PU per clause in the language sample was below 20% (Restrepo, 1998); and (d) the non-verbal IQ score was 80 or above on the KABC-II.

Children with DLD met the following criteria: (a) 4-year-old children scored at or below the cut score of 50 on the morphosyntax subtest of BESA; 5- and 6-year-old children scored below one standard deviation from the mean on the CELF-4 Spanish because it has higher sensitivity than BESA for this age range; (b) scored below the 16th percentile on the TPL screening test; (c) the PU per clause in a language sample was 20% or above; (d) the non-verbal IQ score was 80 or above on the KABC-II; and (e) two native Spanish-speaking speech-language pathologists (SLPs) with more than 15 years of experience confirmed the diagnosis, based on observations at the school setting and during the sessions.

In addition, the children’s parents completed a parental questionnaire that asked about their language concerns, medical and language history, educational level of both the parents and child, relatives with a history of language difficulties, as well as the social and cultural activities that parents engaged in daily with their child (see Supplementary Appendix 1).

### 2.3. Instruments

The analyzed data were obtained from the administration of the questionnaire:

#### 2.3.1. Parental questionnaire (PQ)

The questionnaire consisted of two parts. The first part, known as the parental linguistic concern questionnaire (PLCQ), included eight “Yes” or “No” questions adapted from Restrepo’s (1998) original questions. In each question, parents had space to complete their responses with a brief description of what they observed: 1. Are you concerned about the language of your child? 2. Do other people have difficulty understanding your child? 3. Does your child talk as well as other children of the same age? 4. Does your child speak “funny” or “weird?” 5. Has a family member/teacher commented that your child talks little or talks poorly? 6. Does your child understand most of what is said to him/her? 7. Do you have to repeat what you say to your child more than to other children of the same age? 8. Compared to other children of the same age, does your child have difficulties understanding questions? For each question about their perception of their child’s language, parents’ responses were recorded. If the response indicated parental concern, it was labeled as “risk perception,” which included “yes” responses to questions 1, 2, 4, 5, 7, and 8, as well as “no” responses to questions 3 and 6. Otherwise, the response was recorded as “no risk perception.” The number of risk perceptions for each question was then calculated cumulatively.

The second part of the questionnaire is called the biological and environmental conditions questionnaire (BECQ). In this part, eleven questions were included: 9. the sex of the child; 10. motor problems; 11. neurological problems; 12. psychological problems; 13. the age of production of first words; 14. years of maternal education; 15. years of paternal education; 16. family history of language problems; 17. time children attended preschool; 18. time dedicated to social interaction with children (e.g., playing with toys, doing puzzles); 19. time dedicated to communicative interaction with children (e.g., reading books, talking about daily experiences), and 20. time spent on screens, which are variables that have been discussed in the literature that may influence DLD (Peñaloza, 2018). A complete Spanish version of the PQ is available in Supplementary Appendix 1 as well as the short Spanish version in Supplementary Appendix 2.

### 2.4. Statistical analysis

The following procedure was used for the statistical analysis.

First, the characteristics of the two groups of children, DLD and TLD (henceforth called children from both clinical conditions), were described according to the age groups of 4-, 5-, and 6-year-olds. Differences in categorical data between both clinical conditions were tested by Pearson’s χ^2^ test. Fisher’s exact test and its extension methods were not used due to the design, in which the frequencies in the contingency table were not fixed (Kroonenberg and Verbeek, 2018). To determine differences in continuous data between groups, we performed Welch’s *t*-test, where *P* < 0.05 was interpreted as statistically significant. Non-parametric tests, such as the Mann–Whitney U-test, were not employed as it is recognized that they may produce unreliable *P*-values if the assumption of homoscedasticity is violated (Grimes and Schulz, 2005). We provided 95% confidence intervals for odds ratios and AUC estimates. Effect sizes for continuous variables were calculated using Cohen’s d, and effect sizes for categorical variables were expressed as Phi index or odds ratios. The interpretation of Cohen’s d and Phi index was as follows: null < 0.2; 0.2 ≤ small < 0.5; 0.5 ≤ medium < 0.8; 0.8 ≤ large for continuous variables, and null < 0.1; 0.1 ≤ small < 0.3; 0.3 ≤ medium < 0.5; 0.5 ≤ large for categorical variables (Cohen, 1988).

The distribution of responses to the eight questions in the PLCQ, the first part of the PQ, was compared between the two clinical conditions. Following this comparison, the independent and dependent variables were interchanged, and multiple logistic regression models were created to identify variables that distinguish between the clinical conditions (Knottnerus et al., 2008). Variable selection for the logistic regression models was conducted using a stepwise method based on the Akaike information criterion (AIC). As indicated in the section “3. Results,” statistical analysis using the overall sample of 4–6-year-olds (*n* = 680) allowed us to reduce the number of questions from 8 to 4. The questions thus obtained were used as a four-item PLCQ, a screening tool that yields a score from 0 to 4 according to the number of questions selected by each respondent. The likelihood ratio (LR) is the ratio of the “proportion of true positives” to the “proportion of false positives” (LR+), or the “proportion of false negatives” to the “proportion of true negatives” (LR−). Stratum-specific likelihood ratio (SSLR) was calculated for each of the five strata corresponding to these scores. The weights derived from the LR and SSLR were used to determine the change in pretest and posttest probabilities. The same analysis was conducted for the three age groups. Furthermore, the data of 128 children with no missing responses in the 11 questions of the BECQ, the second part of the PQ, were analyzed to investigate if the biological and environmental information improves the diagnostic performance of the PLCQ. The distribution of the 11 variables was compared between the clinical conditions, and a multiple logistic regression model was constructed to identify questions useful in identifying children with DLD, by interchanging independent and dependent variables. The utility of a diagnostic test can be evaluated by how much the results of the test change the probability of a particular condition expected in an individual, such as the presence or absence of a condition or a property aimed at diagnosing a condition. The LR approach to diagnostic test utility studies uses LR and SSLR as algebraic weighting factors based on Bayes’ theorem to update pretest probabilities into posttest probabilities. Likelihood ratios are typically interpreted as follows: an LR of 10 or greater (or its inverse less than 0.1) indicates a large change between pretest and posttest probabilities; an LR of 5–10 (or its inverse 0.2–0.1) indicates a moderate change between pretest and posttest probabilities; an LR of 2–5 (or its inverse 0.5–0.2) indicates a small change between pretest and posttest probabilities; an LR less than 2 (or its reciprocal 0.5 or greater) indicates no change between pretest and posttest probabilities.

Statistical analysis was performed using the free software environment r (R Core Team, 2021); r was used with RStudio (R Studio Team, 2020) and the effsize package (Torchiano, 2020).

## 3. Results

Table 1 summarizes the sociodemographic and developmental characteristics of children with DLD and TLD across three age groups (4-, 5-, and 6-year-olds) and compares them. The distribution of sex and age was similar between both clinical conditions across all age groups, with almost zero effect sizes. Differences in maternal education were found between DLD and TLD groups in the 5- and 6-year-old age groups, with statistically significant or nearly significant *p*-values, but effect sizes were small for both. Statistically significant differences were observed between clinical conditions in the KABC-II scores in the 4- and 6-year-old age groups, with small and medium effect sizes, respectively. However, these scores were within the normal range for both clinical conditions, and their standard deviations were expected. In contrast, large differences were observed in language parameters measured by BESA (age 4) or CELF-4 (ages 5 and 6) and the PU across all age groups, as expected.

**TABLE 1:** Demographic, sociocultural, and clinical characteristics of children with DLD and TLD in each of the three age groups.

Variable	DLD	TLD	ES	*P*-value
4-year-old [*n* = 240; DLD: *n* = 84 (35%), TLD: *n* = 156 (65%)]				
**Demographic and sociocultural**				
Age (month)	53.0 (3.7)	53.2 (3.2)	0.06^N^	0.200
Sex (female)	35 (42%)	77 (49%)	0.07^N^	0.255
Maternal education (years) (n_DLD_ = 80; n_TLD_ = 150)	11.7 (3.8)	12.3 (4.1)	0.15^N^	0.235
**Cognitive and language development**				
KABC-II (score) (n_DLD_ = 77; n_TLD_ = 123)	100.3 (9.6)	104.3 (10.6)	0.39^S^	0.007
BESA (%)	50.8 (19.0)	83.8 (10.1)	2.51^L^	< 0.001
PU (%)	35.2 (22.5)	13.6 (13.0)	1.37^L^	< 0.001
**5-year-old [*n* = 225; DLD: *n* = 59 (26%), TLD: *n* = 166 (74%)]**				
**Demographic and sociocultural**				
Age (month)	65.4 (3.3)	65.7 (3.5)	0.09^N^	0.587
Sex (female)	21 (36%)	67 (40%)	0.04^N^	0.519
Maternal education (years) (n_DLD_ = 56; n_TLD_ = 162)	10.9 (3.6)	12.4 (4.0)	0.38^S^	0.012
**Cognitive and language development**				
KABC-II (score) (n_DLD_ = 55; n_TLD_ = 124)	100.3 (9.6)	101.3 (11.0)	0.09^N^	0.520
CELF-SR (score: 1–18)	8.6 (2.9)	13.1 (2.2)	1.91^L^	< 0.001
CELF-WS (score: 1–18)	7.6 (2.9)	12.7 (2.6)	1.92^L^	< 0.001
PU (%)	41.3 (22.2)	12.8 (9.8)	2.24^L^	< 0.001
**6-year-old [*n* = 215; DLD: *n* = 42 (19%), TLD: *n* = 173 (80%)]**				
**Demographic and sociocultural**				
Age (month)	77.0 (3.4)	77.5 (3.8)	0.13^N^	0.459
Sex (female)	13 (31%)	74 (43%)	0.10^N^	0.161
Maternal education (years) (n_DLD_ = 41; n_TLD_ = 164)	9.5 (4.1)	10.9 (3.8)	0.36^S^	0.054
**Cognitive and language development**				
KABC-II (score) (n_DLD_ = 42; n_TLD_ = 125)	98.4 (10.1)	104.5 (10.7)	0.58^M^	0.001
CELF-SR (score: 1–18)	7.4 (2.8)	12.6 (2.3)	2.11^L^	< 0.001
CELF-WS (score: 1–18)	6.7 (3.0)	12.2 (2.2)	2.30^L^	< 0.001
PU (%)	44.0 (24.4)	12.7 (9.6)	2.29^L^	< 0.001

Data were summarized by mean (standard deviation), except the variable “sex” reported in the number of cases (percentage). ES, effect size.

To determine whether the eight questions in the PLCQ are effective in identifying children with DLD, we compared the proportion of parental concern by examining the number of positive responses to each question. As presented in Table 2, parents of children with DLD expressed a higher percentage of concern for all questions, although the effect size varied depending on the question. Questions 1, 2, and 5 showed a “medium” effect size, while questions 3, 4, 7, and 8 had a “small” effect size. Question 6, on the other hand, had a null effect size.

**TABLE 2:** Eight questions in the PLCQ in both clinical conditions.

Questions in PLCQ (*n* = 680)	DLD	TLD	ES	*P*-value
1. Are you concerned about the way your child talks?	139 (75%)	183 (37%)	0.34^M^	< 0.001
2. Do other people have difficulty understanding your child?	120 (65%)	94 (19%)	0.44^M^	< 0.001
3. Does your child talk as well as other children of the same age?	105 (57%)	180 (36%)	0.18^S^	< 0.001
4. Does your child speak “funny” or “weird?”	90 (49%)	101 (20%)	0.28^S^	< 0.001
5. Has a family member/teacher commented that your child talks little or talks poorly?	118 (64%)	92 (19%)	0.44^M^	< 0.001
6. Does your child understand most of what is said to him/her?	69 (37%)	147 (30%)	0.07^N^	0.058
7. Do you have to repeat what you say to your child more than to other children of the same age?	75 (41%)	80 (16%)	0.26^S^	< 0.001
8. Compared to other children of the same age, does your child have difficulties understanding questions?	61 (33%)	47 (9%)	0.29^S^	< 0.001

Developmental language disorder_DLD_ and typical language development_TLD_: sample size of both clinical groups, respectively. Data were summarized by the number of cases (percentage). ES, effect size.

Next, a multiple logistic regression model was constructed with the eight questions in the PLCQ as explanatory variables, and clinical conditions (DLD/TLD) as the criterion variable, to identify the questions that contribute to the prediction of DLD. Table 3 shows four questions (1, 2, 5, and 8) selected by the best fit logistic regression model (for groups 4- to 6-year-olds, see Supplementary Appendix 3).

**TABLE 3:** The best fit logistic regression model with four questions in the parental questionnaire.

Model terms	β (SE)	χ^2^	*P*-value	OR (95%CI)
**Global: 4 to 6-year-old (*n* = 680)**				
Best fit model: χ2 (d.f. 4) = 172.43, *P* < 0.001, AIC = 633.63, AUC = 0.795				
Intercept	−2.26 (0.17)	172.57	< 0.001	
1. Are you concerned about the way your child talks?	0.48 (0.25)	3.66	0.056	1.61 (0.99, 2.62)
2. Do other people have difficulty understanding your child?	1.06 (0.25)	18.16	< 0.001	2.90 (1.78, 4.73)
5. Has a family member/teacher commented that your child talks little or talks poorly?	1.17 (0.23)	25.63	< 0.001	3.21 (2.04, 5.05)
8. Compared to other children of the same age, does your child have difficulties understanding questions?	0.64 (0.26)	6.20	0.013	1.90 (1.15, 3.14)

β (SE): regression coefficient (standard error). χ2, Wald’s χ2; OR (95% CI), odds ratio (95% confidence interval).

**FIGURE 1:**
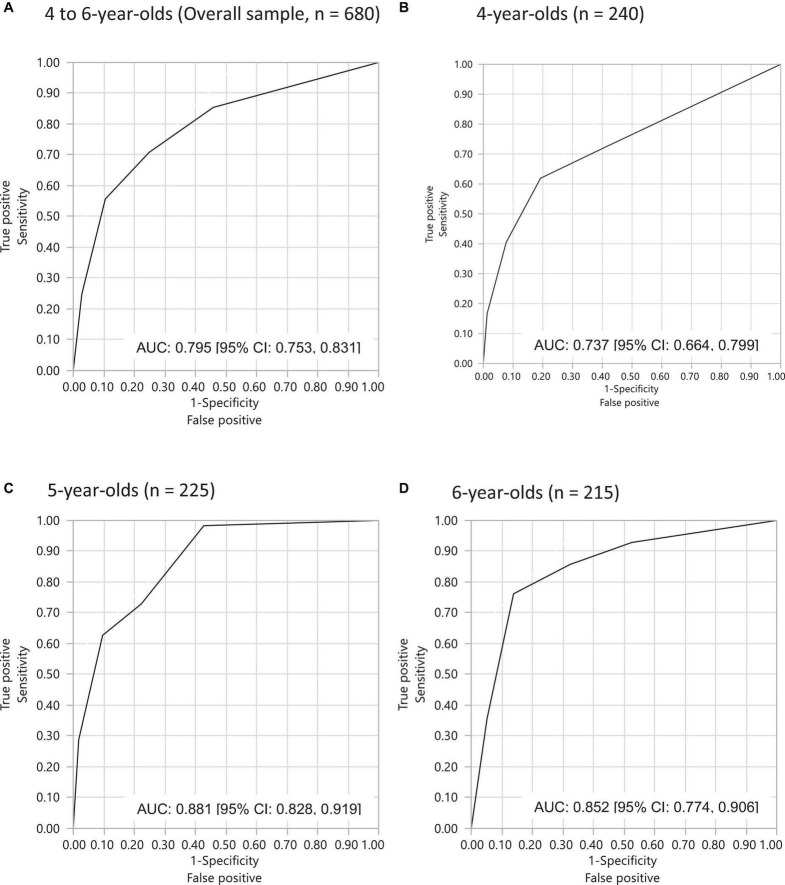
ROC (receiver operating characteristic) curves showing DLD diagnostic accuracy of the PLCQ based on four questions: **(A)** 4- to 6-years-olds (overall sample, *n* = 680); **(B)** 4-year-olds (*n* = 240); **(C)** 5-year-olds (*n* = 225); **(D)** 6-year-olds (*n* = 215).

In addition, ROC curves were generated based on the same multiple logistic regression model. In this, the area under the curve (AUC) was 0.795 (95% CI: 0.753, 0.831). By age, the AUC was 0.737 (95% CI: 0.664, 0.799); 0.881 (95% CI: 0.828, 0.919); 0.852 (95% CI: 0.774, 0.906) for the 4, 5 and 6-year-old samples, respectively, all indicating satisfactory diagnostic performance (See Figure 1).

Table 4 shows the distribution of absolute frequencies of the number of positive responses of parents of DLD and TLD children to the four questions in the PLCQ for all 4- to 6-year-olds (*n* = 680). The SSLR was obtained, as well as the change in the pretest to posttest DLD probability. Here we used 0.12, an estimate of the prevalence of DLD among 4- to 6-year-olds in Mexico obtained in a study in preparation (Auza et al., 2018). This estimate was used as the pretest probability of administering the PLCQ; the posttest probability with SSLR was calculated as a weight factor. The results showed that if a parent reported two out of four concerns, the posttest probability was almost the same as the pretest probability. However, if the parent reported three out of four concerns, the probability of the child having DLD almost tripled, increasing from 0.12 to 0.35. If all four concerns were present, the posttest probability increased more than 4.5 times, from 0.12 to 0.55. On the other hand, the pretest probability of DLD decreased three times to 0.09 when only one concern was reported, and one-third to 0.04 when no concerns were reported. The above analysis was also conducted for each age group of 4-, 5-, and 6-year-olds, with similar results among them (Table 6 in Supplementary Appendix 3).

**TABLE 4:** Stratum-specific likelihood ratio (SSLR) and the change from pretest probability to posttest probability of DLD in the three age groups with four questions in PLCQ.

Stratum	DLD	TLD	SSLR	Pretest probability*	Pretest odds	Posttest odds	Posttest probability
**Global, 4 to 6-year-old (*n* = 680)**							
4	46	14	8.792	0.12	0.136	1.199	0.55
3	57	38	4.014			0.547	0.35
2	28	71	1.055			0.144	0.13
1	27	104	0.695			0.095	0.09
0	27	268	0.270			0.037	0.04
Total	185	495					

SSLR, stratum-specific likelihood ratio.

*For the pretest probabilities, we used estimates of the prevalence of DLD in Mexican children aged 4 to 6 years obtained from another study [Auza, A., Murata, C., and Méndez, I. (under review). Prevalence of developmental language disorders in Mexico. Semin. Speech Lang.].

Data from the BECQ questionnaire were available for 128 cases, representing 19% of the overall sample of 4- to 6-year-olds, 36 (15%) for the 4-year-old group, 45 (20%) for the 5-year-old group, and 47 (22%) for the 6-year-old group. Based on the small sample size and unstable results within each age group, we have presented the overall results from the 128 children. However, for additional information, results by age groups can be found in Table 7 in Supplementary Appendix 3. However, the results of the age-specific analysis are also included in the same table for reference. In both clinical conditions, the distribution of eight questions in the PLCQ in the subgroups was similar to the results obtained from the overall sample. *P*-values were larger in the analysis performed in subgroups, which can be expected *a priori*, because of the reduced power in the statistical tests when reducing the sample size. On the other hand, effect sizes for the eight questions showed a similar pattern between the overall sample and the subgroup of 128 children.

In a multiple logistic regression model for assessing the predictive accuracy of DLD using PLCQ, the following questions were selected: 1, 2, 5, and 8. These questions were found to contribute to the model that best meets the AIC criteria. Its performance was 0.804 (95% confidence interval: 0.683, 0.886) on the AUC-ROC curve. On the other hand, when questions 1, 2, 5, and 8, which comprise the four questions in the PLCQ obtained from the overall sample of 680 subjects, were administered to the same subgroup, the DLD diagnostic performance was 0.808 (95% confidence interval: 0.690, 0.888) on the AUC-ROC curve, almost equal to the best-fit model (Table 8 in Supplementary Appendix 3 and Figure 1). Based on this equivalence, the subgroup of 128 participants was explored to observe whether the addition of the BECQ questions to the PLCQ might improve the performance of the parental questionnaire. To evaluate its performance, first, it was examined whether there were significant differences in the distribution between the clinical groups for each of the eleven questions in BECQ, as we did for the eight questions in the PLCQ. We found moderate or large effect sizes for only two questions, such as 17. *Time children attended preschool*, and 19. *Communicative interaction with children*. Statistically significant differences between DLD and TLD groups were detected for questions 10. *Neurological and/or psychological problems*, 17, and 19. The effect sizes and *P*-values were small (Phi = 0.20), *P* = 0.023 for question 2; large (Cohen’s *d* = 0.20), *P* < 0.001 for question 17; and medium (Cohen’s *d* = 0.54), *P* = 0.023, for question 19 (Table 9 in Supplementary Appendix 3). Second, a multiple logistic regression model was constructed with the BECQ questions as explanatory variables and DLD (or TLD) as the criterion variable, and the best-fit model was searched for, following the AIC criteria; questions 10, 16, 17, and 19 were selected as explanatory variables. However, in this model, the association of questions 10, 16, and 19 with the criterion variables was very low compared to the association shown in question 17. Given so, a logistic regression model with only question 17 as the explanatory variable was created. Then, ROC curves were constructed based on these models. As a result, ROC-AUC was 0.767 (95% CI: 0.634, 0.863) in the best-fit model with four variables. In the single-variable model with only question 17, the result was 0.753 (95% CI: 0.640, 0.840), indicating that both showed almost equal performance. Therefore, based on the parsimony principle, only question 17. time children attended preschool was used as an explanatory variable in the BECQ (Table 10 in Supplementary Appendix 3).

Finally, the predicted probabilities of DLD were obtained as shown in Table 5. Very few cases in this subgroup had a PLCQ score of 4 (2 in the group of DLD and 3 in the group of TLD). Therefore, this stratum of 4 was combined with that of score 3. As a result, SSLRs of 0.653, 0.166, 0.079, and 0.019 were calculated for the score 3–4, score 2, score 1, and score 0 strata, respectively. Regarding the BECQ questions, the only explanatory variable was 17. time children attended preschool. The variable showed a likelihood ratio of 1.829 below the median and 0.525 above the median. When setting the pretest probability of the PLCQ at 0.13, which represents the estimated prevalence of DLD among children aged 4–6 in Mexico, the posttest probability of the PLCQ varied from 0.37 to 0.02, depending on the PLCQ score. Furthermore, the posttest probability of the PLCQ was higher than that of the BECQ. When weighted by likelihood ratios, as pretest probabilities (i.e., the binary preschool enrollment years), the posttest probabilities were 0.52 or 0.24, 0.22 or 0.07, 0.12 or 0.04, and 0.03 or 0.01 for BECQ pretest probabilities of 0.37, 0.13, 0.07, and 0.02, respectively.

**TABLE 5:** DLD probabilities at the pretest and two posttests combined with four questions in the PLCQ and (time children attended preschool) data.

Prob1	Odds 1	PLCQ4	DLD (*n* = 21)	TLD (*n* = 41)	SSLR	Odds 2	Prob 2	Preschool > median	LR	Odds 3	Prob 3
0.12	0.136	3–4	12	14	4.367	0.596	0.37	No	1.829	1.194	0.52
		2	5	23	1.108	0.151	0.13	No	1.829	0.303	0.22
		1	3	29	0.527	0.072	0.07	No	1.829	0.144	0.12
		0	1	41	0.124	0.017	0.02	No	1.829	0.034	0.03
								Yes	0.525	0.342	0.24
								Yes	0.525	0.087	0.07
								Yes	0.525	0.041	0.04
								Yes	0.525	0.010	0.01

Prob 1: estimated prevalence of DLD in Mexico used as the pretest probability for DLD prior to the application of four questions in the PLCQ. Odds 1: pretest odds calculated from pretest probability. PLCQ4: 4 stratums of SSLR based on the results of the four questions in the PLCQ. Odds 2: posttest odds for DLD according to the result of four questions on PLCQ weighted by the SSLR. Prob 2: posttest probability calculated from the posttest odds. Preschool: (time children attended preschool) dichotomized into ≤ and > the median within age groups (for 4-, 5-, and 6-year-olds, 1.5, 2.5, and 3.5 years, respectively). LR: likelihood ratios for children ≤ median or > median for (time children attended preschool). Odds 3: posttest odds for DLD according to (time children attended preschool) weighted by the LR. Prob 3: posttest probability calculated after the combination of 4 questions on PLCQ and (time children attended preschool).

## 4. Discussion

The purpose of this paper was to analyze whether a parental language concern questionnaire can help identify children with developmental language disorder. Our hypothesis was confirmed, as the questionnaire can provide sufficient information and be used as a screening tool to help identify children with DLD. The data presented in this study emphasize the importance of considering parental linguistic concerns as part of a screening process. As previously stated, parents can be valuable allies in obtaining reliable information about children, as they are attuned to their communicative and linguistic needs and difficulties, regardless of their cultural and linguistic backgrounds (Thal et al., 2000; Bishop and McDonald, 2009; Guiberson and Rodríguez, 2010; Peñaloza et al., 2021). This is particularly significant because the absence of suitable screening and assessment tools for certain underrepresented populations has resulted in inaccurate under-diagnosis of children with DLD. Regarding more qualitative information on which questions in the PLCQ have the best predictive value for identifying children with DLD, our hypothesis was also confirmed. We observed that specific questions in the PLCQ better improved the performance of this questionnaire. Some questions, such as your child speaks as well as other children of the same age? and Do you have to repeat a question to your child several times in order for him/her to understand it? were removed from the linguistic parental concern set of questions since they did not contribute to identifying children with DLD. An interesting outcome is that by using a smaller number of more sensitive screening questions, many children at risk of having DLD can be identified, even without administering any tests to them yet. Our previous study (Auza et al., 2023) showed that a combination of a parental questionnaire and a screening test could satisfactorily identify children with DLD. Administering these questions to families was useful as a screening test for identifying DLD in 4-, 5-, and 6-year-olds, as evidenced by the SSLR of the questionnaire. Additionally, our study demonstrated which questions were more helpful and effective in identification. Questions such as Does your child talk as well as other children of the same age? may not be accurate since parents may not always be aware of linguistic developmental milestones and therefore do not have a point of comparison. Similarly, if we ask them Does your child speak “funny” or “weird?” Parents may not always consider that speaking differently, strangely, or funny does not necessarily imply a language problem. This may be due to their limited knowledge of morphosyntactic milestones. They may not be aware of when children start to combine words (emergence of syntax), when they usually start using functional words, and so on. Therefore, parental linguistic concerns during these ages may also be focused on other aspects beyond grammar. Other questions about comparison with other children, such as Do you have to repeat what you say to your child more than to other children of the same age? may be related to parents’ interpretation of their child’s behavior. Parents often understood this question as being distracted or disobedient to commands rather than having difficulty understanding language. Furthermore, statistical analyses indicated that four questions about parental linguistic concern are sufficient as a screening tool, especially in contexts where language pathologists and/or language tests may not be readily available in large clinical or educational settings. When three or more concern questions are obtained in the questionnaire, parents should be encouraged to seek evaluation by a clinician to confirm the diagnosis, as the probability of having DLD increases threefold with these concern questions. Conversely, if there is only one concern or none, it is acceptable to lower suspicions about having DLD.

Our last hypothesis was partially confirmed. We expected that including more biological and environmental questions in the questionnaire would improve its performance, but our results showed only a slight improvement with the inclusion of one question: time children attended preschool. A prolonged and consistent stay in preschool may work as a critical mass in providing children with linguistic tools, such as greater grammatical complexity, as they are exposed to more interactions and varied discursive practices. However, our results only showed a slight improvement in performance with the inclusion of one additional question. Therefore, our study may not provide conclusive evidence on whether other biological or environmental factors, such as the age of onset of first words and family heritability, may also play a significant role, as suggested by several previous studies (Pelchat et al., 2003; Raviv et al., 2004; Bornstein et al., 2007; Farkas et al., 2015). Our study has demonstrated that communicative interaction between parents and their children is a significant factor at the ages of four and six. We have observed medium and large effect sizes, respectively, which suggest that a lower level of communicative interaction may be associated with the diagnosis of DLD. Surprisingly, we have not found any association between low maternal education or age of onset of first words and DLD, possibly due to the limited statistical power in the small subgroup analyzed. However, it is worth noting that the existence of associations does not necessarily imply a predictive value in the parental questionnaire. For instance, previous research has shown that maternal education is linked to DLD in several studies (Hart and Risley, 1995; Hoff, 2003). This association may have an impact on language development, particularly on vocabulary acquisition, but its influence on morphosyntax may be marginal (Abutbul-Oz and Armon-Lotem, 2022). Although most of the biological and environmental questions did not improve the performance of the parental questionnaire, it is still important to identify which variables showed an association and which ones may become associated with a larger sample in future studies. For instance, time spent on screens did not demonstrate a significant association in our analysis, but it had a small effect in the general analysis of the 128 children and a large effect in the 6-year-old group. This suggests that, by increasing the statistical power of our data in a future study, this variable may contribute to predicting which children are at risk of developing DLD. Other variables related to social interaction are also worth considering in future research, even in younger children. For example, it would be interesting to explore the hours parents spend playing with their children, the frequency of doing homework together, and the frequency of shared playtime.

Although several studies have reported associations between various variables and language disorders, few have examined whether these associations improve the predictive validity of parental questionnaires. Our study has contributed to this issue by analyzing some factors that do not enhance the performance of the parental questionnaire, despite being known to influence language disorders. Therefore, we can conclude that using a reduced number of sensitive parental linguistic concern questions is a reliable method for identifying children with language disorders, particularly in settings where standardized assessment instruments or special education services are scarce. Future research could explore the identification of younger children who are at risk of developing DLD, including work with 3-year-olds. Additionally, we believe that these questions are worth exploring in other cultures facing similar issues, in order to generalize our results.

## 5. Limitations of the study

Our study has some limitations that should be noted. Firstly, our findings about the usefulness of some of the biological and environmental questions are inconclusive, as they might not have been clear to some parents, resulting in incomplete answers. Hence, other biological and environmental factors could potentially play a significant role in identifying children with DLD, when answers are not omitted. With larger sample sizes, we may be able to identify additional factors that contribute to the identification of more children with DLD. To address this issue, future studies should ensure that parents answer all questions in the questionnaire or provide improved explanations on how to complete it. In an ideal situation, a clinician should administer the questionnaire to parents, ensuring that all questions are answered accurately. Furthermore, it is important to acknowledge that parents, especially those with lower levels of education, may tend to underestimate certain aspects of their child’s language development, such as grammar use (Caraveo-Anduaga et al., 2002; Keegstra et al., 2007; McLeod and Harrison, 2009). Additionally, it is worth noting that even when there is a family history of language impairments, parents may tend to downplay its significance. Additionally, many parents from our sample report short linguistic and social interactions with their children, that may not be sufficient for optimal language development as reported previously in the literature.

Despite the reduced sample size, our study provides valuable insights for identifying monolingual children with DLD using parental linguistic concern questions.

## Data availability statement

The raw data supporting the conclusions of this article will be made available by the authors, without undue reservation.

## Ethics statement

The studies involving human participants were reviewed and approved by the Comité de Ética en Investigación, Hospital General Dr. Manuel Gea González. Written informed consent to participate in this study was provided by the participants’ legal guardian/next of kin.

## Author contributions

AAB, CM, and CP contributed equally to the design and implementation of the research, to the analysis of the results, and to the writing of the manuscript. AAB and CP were in charge of data collection and coding. CM supervised, supported, and processed statistical analysis. AAB guided this research project from beginning to end. All authors contributed to the article and approved the submitted version.

## Editor’s note

Maria-José Ezeizabarrena edited the article in collaboration with Melita Kovacevic, University of Zagreb, Zagreb, Croatia.
