# Erratum: Predictive validity of a parental questionnaire for identifying children with developmental language disorders

**DOI:** 10.3389/fpsyg.2023.1258680

**Published:** 2023-08-10

**Authors:** Frontiers Production Office

**Affiliations:** Frontiers Media SA, Lausanne, Switzerland

**Keywords:** parental questionnaires, developmental language disorder (DLD), early identification, Spanish-speaking children, parental linguistic concerns

Due to a publishing error, the co-editor accreditation for this article needs to be amended. A note has been added to the original article, as shown below.

**Editor's note**

Maria-José Ezeizabarrena edited the article in collaboration with Melita Kovacevic, University of Zagreb, Zagreb, Croatia.

The publisher apologizes for this mistake. The original article has been updated.

