# Supplementary material for: Predictive validity of a parental questionnaire for identifying children with developmental language disorders
Source: Front Psychol. 2023 Jun 19;14:1110449. doi: 10.3389/fpsyg.2023.1110449 (PMC10316708; doi:10.3389/fpsyg.2023.1110449)
Supplement: Supplementary file 1 [file Presentation_1.pdf]

11. Supplementary material

Appendix 1

PARENTAL QUESTIONNAIRE (PQ)

Spanish version

Este cuestionario consiste en una serie de preguntas sobre la historia de vida de su hijo(a) y de su familia. Le pedimos que las conteste de la manera más completa y verídica posible. Desde ya agradecemos enormemente su colaboración.

Nombre del niño: _______________________________________

Sexo: Masculino ___ Femenino ___

Edad: _______

Fecha de nacimiento: ______________________

Fecha en que completó este cuestionario: ____________________

¿Quiénes contestaron este cuestionario? (mamá, papá, abuela, etc.): _____________________

Teléfono de contacto: _________________

PRIMERA PARTE: PREOCUPACIÓN PARENTAL SOBRE EL LENGUAJE (PLCQ)

**I. Responda las siguientes preguntas en relación con lo que usted observa o percibe del lenguaje de su hijo**	
.1. ¿Le preocupa la manera en que su niño/a habla?	Sí __ NO ___
2. ¿Otras personas tienen dificultad para entender al niño/a?	Sí __ NO ___
3. ¿Su niño habla tan bien como los otros niños de su misma edad?	Sí __ NO ___
4. ¿Su niño habla “chistoso” o “raro”?	Sí __ NO ___
5. ¿Algún familiar / maestro le ha comentado que su niño habla poco o habla mal?	Sí __ NO ___
6. ¿Su niño/a entiende la mayoría de lo que se le dice?	Sí __ NO ___
7. ¿Usted tiene que repetir lo que se le dice a su niño/a más que a otros niños de la misma edad?	Sí __ NO ___

8. Comparado con otros niños de la misma edad, ¿su niño/a tiene problemas para entender preguntas?	Sí __ NO ___

SEGUNDA PARTE: CONDICIONES BIOLÓGICAS Y DEL ENTORNO (BECQ)

II. ANTECEDENTES DEL NIÑO

9. Sexo del niño: _____ niña ______ niño

¿El niño/a ha tenido un desarrollo normal en los siguientes ámbitos?

10. Neurológico -Sí____ -No____ ¿Qué dificultad ha presentado? ___________________________

11. Motor -Sí____ -No____ ¿Qué dificultad ha presentado? ___________________________

12. Psicológico -Sí____ -No____ ¿Qué dificultad ha presentado? ___________________________

13. ¿A qué edad el niño/a dijo sus primeras palabras (distintas a "mamá" y "papá")?

- A los _______________

III. INFORMACIÓN SOBRE LOS PADRES DEL NIÑO

En relación con la mamá y al papá del niño, indique a continuación qué niveles de escolaridad cursó y cuántos años de estudios completó en cada nivel

	**14. Mamá**	**15. Papá**
- Primaria	0 1 2 3 4 5 6	0 1 2 3 4 5 6
- Secundaria	1 2 3	1 2 3
- Preparatoria	1 2 3	1 2 3
- Carrera técnica	1 2 3 4	1 2 3 4
- Carrera universitaria	1 2 3 4 5 6 7	1 2 3 4 5 6 7
- Posgrado	1 2 3 4 5 6 7	1 2 3 4 5 6 7

16. ¿Algún miembro del núcleo familiar (hermanos, papá, mamá, tíos, abuelos) presentó problemas de lenguaje en su proceso de aprendizaje?

IV. INTERACCIÓN FAMILIAR Y EXPERIENCIAS DEL NIÑO

17. El niño ¿Asiste actualmente o asistió a un preescolar? -Sí____ -No____

Si el niño va o fue a un preescolar, ¿a qué edad empezó a asistir? A los ______ años

18-19. En relación con el cuidador principal del niño, ¿qué tan frecuentemente realiza con el niño estas acciones? La última semana hizo estas actividades con su hijo

	Todos o casi todos los días	Al menos una vez a la semana	Al menos una vez al mes	Nunca o casi nunca
- Hacer las tareas juntos				
- Armar un rompecabezas juntos				
- Ver televisión juntos				
- Conversar sobre las experiencias diarias del niño, sus compañeros, etc.				
- Leer un libro (de cuentos, de historietas, etc.) juntos.				

20. ¿Cuántas horas diarias, aproximadamente, pasa la televisión encendida en el hogar/horas o el niño frente a pantallas? _______ horas.

¡¡MUCHAS GRACIAS POR SU COLABORACIÓN!

Appendix 2.
Short-version of the PQ

Spanish version

Este cuestionario consiste en una serie de preguntas sobre la historia de vida de su hijo(a) y de su familia. Le pedimos que las conteste de la manera más completa y verídica posible. Desde ya agradecemos enormemente su colaboración.

Nombre del niño: _______________________________________

Sexo: Masculino ___ Femenino ___

Edad: _______ Fecha de nacimiento: ______________________

Fecha en que completó este cuestionario: ____________________

¿Quiénes contestaron este cuestionario? (mamá, papá, abuela, etc.): _____________________

Teléfono de contacto: _________________

PRIMERA PARTE: PREOCUPACIÓN PARENTAL SOBRE EL LENGUAJE

**I. Responda las siguientes preguntas en relación con lo que usted observa o percibe del lenguaje de su hijo**	
1. ¿Le preocupa la manera en que su niño/a habla?	Sí ____ No ____
2. ¿Otras personas tienen dificultad para entender al niño/a?	Sí ____ No ____
5. ¿Algún familiar / maestro le ha comentado que su niño habla poco o habla mal?	Sí ____ No ____
8. Comparado con otros niños de la misma edad, ¿su niño/a tiene problemas para entender preguntas?	Sí ____ No ____

SEGUNDA PARTE: EXPERIENCIAS DE INTERACCIÓN DEL NIÑO

**17. El niño ¿asiste actualmente o asistió a un preescolar?**	Sí ____ No ____
Si el niño va o fue a un preescolar, ¿A qué edad empezó a asistir?	A los ______ años

Appendix 3.
Supplementary Tables

Table 6. Number of children in four questions PLCQ in b in each sample of 4-, 5- oth clinical conditions and the change from pretest to posttest probabilities of DLD calculated using SSLR.

Stratum	DLD	TLD	SSLR	**Pretest probability** *	Pretest odds	Posttest odds	**Posttest probabilit**
**4-year-olds (n = 240)**							
4	14	2	13.000	**0.14**	0.163	2.116	**0.68**
3	20	10	3.714			0.605	**0.38**
2	18	18	1.857			0.302	**0.23**
1	9	35	0.478			0.078	**0.07**
0	23	91	0.469			0.076	**0.07**
Total	84	156					
**5-year-olds (n = 225)**							
4	17	3	15.944	**0.11**	0.124	1.971	**0.66**
3	20	13	4.329			0.535	**0.35**
2	6	21	0.804			0.099	**0.09**
1	15	34	1.241			0.153	**0.13**
0	1	95	0.030			0.004	**0.004**
Total	59	166					
**6-year-olds (n = 215)**							
4	15	9	6.865	**0.11**	0.124	0.848	**0.46**
3	17	5	4.668			0.577	**0.37**
2	4	32	0.515			0.064	**0.06**
1	3	35	0.353			0.044	**0.04**
0	3	82	0.151			0.019	**0.02**
Total	42	173					

SSLR: Stratum-Specific Likelihood Ratio. *We used as pretest probabilities point estimates of the prevalence of DLD among 4-, 5-, and 6-year-old children in Mexico [52].

Table 7. Comparison of the eight PLCQ questions between both clinical conditions in a subgroup of 128 children with no missing values

Eight questions in 1st part of PLCQ	DLD	TLD	ES	P-value
**Global: 4 to 6-year-old (n = 128; DLD: n = 21 [16%], TLD: n = 107 [84%])**				
1. Are you concerned about the way your child talks?	18 (86%)	54 (50%)	0.26^{S}	0.003
2. Do other people have difficulty understanding your child?	14 (67%)	27 (25%)	0.33^{M}	<0.001

3.	Does your child talk as well as other children of the same age?	14 (67%)	34 (32%)	0.27^S	0.003
4.	Does your child speak "funny" or "weird"?	8 (38%)	22 (21%)	0.15^S	0.083
5.	Has a family member/teacher commented that your child talks little or talks poorly?	14 (67%)	24 (22%)	0.36^M	<0.001
6.	Does your child understand most of what is said to him/her	7 (33%)	24 (22%)	0.10^N	0.286
7.	Do you have to repeat what you say to your child more than to other children of the same age?	13 (62%)	24 (22%)	0.32^M	<0.001
8.	Compared to other children of the same age, does your child have difficulties understanding questions?	5 (24%)	15 (14%)	0.10^N	0.259
**4-year-old (n = 36; DLD: n = 10 [28%], TLD: n = 26 [72%])**					
1.	Are you concerned about the way your child talks?	8 (80%)	15 (58%)	0.21^S	0.212
2.	Do other people have difficulty understanding your child?	5 (50%)	9 (35%)	0.14^S	0.396
3.	Does your child talk as well as other children of the same age?	7 (70%)	10 (38%)	0.28^S	0.090
4.	Does your child speak "funny" or "weird"?	5 (50%)	6 (23%)	0.26^S	0.116
5.	Has a family member/teacher commented that your child talks little or talks poorly?	7 (70%)	7 (27%)	0.40^M	0.018
6.	Does your child understand most of what is said to him/her	3 (30%)	5 (19%)	0.12^S	0.486
7.	Do you have to repeat what you say to your child more than to other children of the same age?	7 (70%)	8 (31%)	0.36^M	0.033
8.	Compared to other children of the same age, does your child have difficulties understanding questions?	2 (20%)	3 (12%)	0.11^S	0.511

Data were summarized by number of cases (percentage). ES: Effect Size.

Table 7. (continued)

Eight questions in 1st part of PLCQ		DLD	TLD	ES	P-value
**5-year-old (n = 45; DLD: n = 7 [16%], TLD: n = 38 [84%])**					
1.	Are you concerned about the way your child talks?	7 (100%)	14 (37%)	0.46^M	0.002
2.	Do other people have difficulty understanding your child?	5 (71%)	3 (8%)	0.60^L	<0.001
3.	Does your child talk as well as other children of the same age?	5 (71%)	5 (13%)	0.51^L	<0.001
4.	Does your child speak "funny" or "weird"?	0 (0%)	4 (11%)	0.14^S	0.369

5.	Has a family member/teacher commented that your child talks little or talks poorly?	6 (86%)	3 (8%)	0.71^{L}	<0.001
6.	Does your child understand most of what is said to him/her	1 (14%)	3 (8%)	0.08^{N}	0.585
7.	Do you have to repeat what you say to your child more than to other children of the same age?	4 (57%)	2 (5%)	0.55^{L}	<0.001
8.	Compared to other children of the same age, does your child have difficulties understanding questions?	2 (29%)	3 (8%)	0.24^{S}	0.110
**6-year-old (n = 47; DLD: n = 4 [9%], TLD: n = 43 [91%])**					
1.	Are you concerned about the way your child talks?	3 (75%)	25 (58%)	0.10^{N}	0.511
2.	Do other people have difficulty understanding your child?	4 (100%)	15 (35%)	0.37^{M}	0.011
3.	Does your child talk as well as other children of the same age?	2 (50%)	19 (44%)	0.03^{N}	0.823
4.	Does your child speak "funny" or "weird"?	3 (75%)	12 (28%)	0.28^{S}	0.053
5.	Has a family member/teacher commented that your child talks little or talks poorly?	1 (25%)	14 (33%)	0.05^{N}	0.756
6.	Does your child understand most of what is said to him/her	3 (75%)	16 (37%)	0.22^{S}	0.141
7.	Do you have to repeat what you say to your child more than to other children of the same age?	2 (50%)	14 (33%)	0.10^{S}	0.481
8.	Compared to other children of the same age, does your child have difficulties understanding questions?	1 (25%)	9 (21%)	0.03^{N}	0.849

n_{DLD} and n_{TLD}: sample size of the DLD group and TLD group, respectively.
Data were summarized by number of cases (percentage). ES: Effect Size.

Table 8. Comparison of the original eight questions of concern in a subgroup of 128 children with no missing values in the PLCQ, and the model based on four questions of concern from 680 children.

Model terms	β (SE)	χ^2	P-value	OR [95%CI]
**Global: 4 to 6-year-old (n = 128)**				
Model 1, best fit model: χ2 (d.f. 3)= 23.06, P <0.001, AIC = 99.53, AUC = 0.804 [95% CI: 0.683, 0.886]				
Intercept	-2.26 (0.17)	172.57	< 0.001	
2. Do other people have difficulty understanding your child?	1.09 (0.58)	3.61	0.057	2.99 [0.97, 9.24]

5. Has a family member/teacher commented that your child talks little or talks poorly?	1.26 (0.58)	4.63	0.031	3.51 1.12, 11.01]
8. Do you have to repeat what you say to your child more than to other children of the same age?	0.87 (0.59)	2.20	0.141	2.39 [0.76, 7.55]
Model 2, consisted of questions 1, 2, 5 and 8: χ2 (d.f. 4) = 22.99, P <0.001, AIC = 101.77, AUC = 0.808 [95% CI: 0.690, 0.888]				
Intercept	-3.43 (0.67)	26.36	<0.001	
1. Are you concerned about the way your child talks?	0.99 (0.72)	1.92	0.166	2.70 [0.66, 11.04]
2. Do other people have difficulty understanding your child?	1.07 (0.57)	3.46	0.063	2.91 [0.94, 8.98]
5. Has a family member/teacher commented that your child talks little or talks poorly?	1.33 (0.56)	7.14	0.018	3.80 [1.26, 11.43]
8. Compared to other children of the same age, does your child have difficulties understanding questions?	0.15 (0.66)	0.05	0.917	1.16 [0.32, 4.20]

Table 8. (continued)

Model terms	β (SE)	χ^2	P-value	OR [95%CI]
**4-year-olds (n = 36)**				
Modelo 1, best fit model: χ2 (d.f. 1) = 5.61, P = 0.018, AIC = 41.30, AUC = 0.715 [95% CI: 0.518, 0.855]				
Intercept	-1.85 (0.62)	8.83	0.0013	
5. Has a family member/teacher commented that your child talks little or talks poorly?	1.85 (0.82)	5.07	0.024	6.33 [1.27, 31.57]
Model 2, consisted of questions 1, 2, 5 and 8: χ2 (d.f. 4) = 7.03, P 0.135, AIC = 47.51, AUC = 0.754 [95% CI: 0.548, 0.886]				
Intercept	-2.32 (0.97)	5.73	0.017	

1. Are you concerned about the way your child talks?	0.71 (1.03)	0.87	0.493	2.03 [0.27, 15.42]
2. Do other people have difficulty understanding your child?	-0.84 (1.08)	0.60	0.438	0.43 [0.05, 3.58]
5. Has a family member/teacher commented that your child talks little or talks poorly?	2.16 (1.07)	4.09	0.043	8.69 [1.07, 70.57]
8. Compared to other children of the same age, does your child have difficulties understanding questions?	1.07 (1.15)	0.87	0.351	2.93 [0.31, 27.97]
**5-year-olds (n = 36)**				
Modelo 1, best fit model: χ2 (d.f. 2) = 25.95, P < 0.001, AIC = 19.53, AUC = 0.961 [95% CI: 0.834, 0.992]				
Intercept	-19.97 (1624)*	0.00	0.990	
1. Are you concerned about the way your child talks?	4.36 (1.50)	8.46	0.004	78.0 [4.1, 1469.2]
5. Has a family member/teacher commented that your child talks little or talks poorly?	17.40 (1624)*	0.00	0.992	3.6E8 (0, ---)

Table 8. (continued)

Model terms	β (SE)	χ^2	P-value	OR [95%CI]
*Model 2, consisted of questions 1, 2, 5 and 8***:** χ2 (d.f. 4) = 26.73, P < 0.001, AIC = 23.71, AUC = 0.962 [95% CI: 0.779, 0.995]				
Intercept	-19.99 (1607)*	0.00	0.990	
1. Are you concerned about the way your child talks?	16.99 (1607)*	0.00	0.992	2.4E8 [0, ---]
2. Do other people have difficulty understanding your child?	1.20 (1.55)	0.60	0.438	3.33 [0.16, 69.56]
5. Has a family member/teacher commented that your child talks little or talks poorly?	3.91 (1.54)	6.43	0.011	50.0 [2.4, 1026.6]

8. Compared to other children of the same age, does your child have difficulties understanding questions?	0.82 (2.45)	0.11	0.738	2.27 [0.02, 278.47]
**6-year-olds (n = 47)**				
Modelo 1, best fit model: χ^2 (d.f. 1) = 7.80, P = 0.005, AIC = 23.83, AUC = 0.826 [95% CI: 0.742, 0.887]				
Intercept	-18.2 (1695)*	0.00	0.991	
2. Do other people have difficulty understanding your child?	16.88 (1695)*	0.00	0.992	2.1E8 [0, ---]
Model 2, consisted of questions 1, 2, 5 and 8: χ^2 (d.f. 4) = 8.16, P = 0.086, AIC = 30.67, AUC = 0.866 [95% CI: 0.650, 0.958]				
Intercept	-17.98 (1683)*	0.00	0.992	
1. Are you concerned about the way your child talks?	-0.20 (1.34)	0.02	0.879	0.82 [0.06, 11.25]
2. Do other people have difficulty understanding your child?	17.0 (11683)*	0.00	0.992	2.4E8 [0, ---]
5. Has a family member/teacher commented that your child talks little or talks poorly?	-0.71 (1.34)	0.28	0.594	0.49 [0.04, 6.74]
8. Compared to other children of the same age, does your child have difficulties understanding questions?	0.14 (1.37)	0.01	0.916	1.16 [0.08, 16.96]

Table 9. Distribution of the 11 variables in BECQ, the second part of the PQ, between the clinical groups.

11 variables of BECQ	DLD	TLD	ES	P-value
Overall sample: 4-, 5- and 6-year-old (n = 128; DLD: n = 21 [16%], TLD: n = 107 [84%])				
1. Sex of the children [female]	9 (43%)	45 (42%)	0.02^{N}	0.946
2. Motor problems [yes]	0 (0%)	0 (0%)	---	---
3. Neurological and psychological problems [yes]	1 (5%)	0 (0%)	0.20^{S}	0.023
4. Age of first words [month]	12 (14.5)	12(8)	0.13^{N}	0.395
5. Time children attended preschool [year]	1.5 (2)	2.5 (2)	0.99^{L}	<0.001
6. Maternal education [year]	12 (5)	12 (7)	0.24^{S}	0.237

7.	Paternal education [year]	9 (4)	11 (6)	0.20^{S}	0.301
8.	Family history of language problems [yes]	6 (29%)	31 (29%)	0.00^{N}	0.971
9.	Time spent on screens [hour]	4 (5)	3 (3)	0.45^{S}	0.133
10.	Social interaction with children [Score]	13 (2)	13 (3)	0.23^{S}	0.248
11.	Communicative interaction with children [Score]	10 (3)	8 (3)	0.54^{M}	0.023
4-year-old (n = 36; DLD: n = 10 [28%], TLD: n = 26 [72%])					
1.	Sex of the children [female]	5 (50%)	8 (31%)	0.18^{S}	0.282
2.	Motor problems [yes]	0 (0%)	0 (0%)	---	---
3.	Neurological and psychological problems [yes]	1 (10%)	0 (0%)	0.27^{S}	0.102
4.	Age of first words [month]	12 (16)	12(6.8)	0.45^{S}	0.390
5.	Time attending preschool [year]	0.5 (1.0)	1.5 (0.5)	0.90^{L}	0.008
6.	Maternal education [year]	12.5 (6.3)	13 (7.8)	0.37^{S}	0.236
7.	Paternal education [year]	10.5 (4.3)	9.5 (7.5)	0.24^{S}	0.368
8.	Family history of language problems [yes]	4 (40%)	11 (42%)	0.02^{N}	0.900
9.	Time spent on screens [hour]	4 (6.5)	4 (4)	0.31^{S}	0.514
10.	Social interaction with children [Score]	13 (2)	13 (3.3)	0.00^{N}	1.000
11.	Communicative interaction with children [Score]	10 (3)	8 (2.5)	0.58^{M}	0.079
5-year-old (n = 45; DLD: n = 7 [16%], TLD: n = 38 [84%])					
1.	Sex of the children [female]	3 (43%)	18 (47%)	0.04^{N}	0.826
2.	Motor problems [yes]	0 (0%)	0 (0%)	0.00^{N}	---
3.	Neurological and psychological problems [yes]	0 (0%)	0 (0%)	0.00^{N}	---
4.	Age of first words [month]	12 (13)	12(7.5)	0.07^{N}	0.874
5.	Time attending preschool [year]	1.5 (1.0)	2.5 (1.0)	0.38^{S}	0.232
6.	Maternal education [year]	11 (3)	12 (7)	0.37^{S}	0.184
7.	Paternal education [year]	9 (4)	12 (7)	0.45^{S}	0.125
8.	Family history of language problems [yes]	2 (29%)	10 (26%)	0.02^{N}	0.300
9.	Time spent on screens [hour]	3 (2)	3 (3)	0.11^{N}	0.284
10.	Social interaction with children [Score]	14 (4)	13 (2.5)	0.27^{S}	0.516
11.	Communicative interaction with children [Score]	8 (5)	8.5 (3)	0.06^{N}	0.492

6-year-old (n = 47; DLD: n = 4 [9%], TLD: n = 43 [91%])

1.	Sex of the children [female]	1 (25%)	19 (44%)	0.11[S]	0.338
2.	Motor problems [yes]	0 (0%)	0 (0%)	0.00[N]	---
3.	Neurological and psychological problems [yes]	0 (0%)	0 (0%)	0.00[N]	---
4.	Age of first words [month]	13 (13.8)	12(8)	0.07[N]	0.896
5.	Time attending preschool [year]	2.5 (1.5)	3.5 (1.0)	1.52[L]	0.047
6.	Maternal education [year]	9 (8.3)	12 (4)	0.51[M]	0.460
7.	Paternal education [year]	9 (8.3)	12 (3)	0.23[S]	0.788
8.	Family history of language problems [yes]	0 (0%)	10 (23%)	0.16[S]	0.918
9.	Time spent on screens [hour]	6 (6.3)	3 (3)	1.14[L]	0.211
10.	Social interaction with children [Score]	13 (5.8)	12 (4)	0.29[S]	0.666
11.	Communicative interaction with children [Score]	10 (2.3)	8 (2)	1.09[L]	0.035

n Developmental Language Disorders. n Typical Language Development: sample size of both clinical groups, respectively.
Data were summarized by number of cases (percentage) or median (Interquartile range).

Table 10. Logistic regression models for the prediction of DLD by PLCQ Part II items, obtained by variable selection based on AIC, for children aged 4-6 years overall and each age group.

Questions in PLCQ		β (SE)	χ^2	P-value	OR [95%CI]
4 to 6-year-old (n = 128)					
Model 1. Best fit model (n = 128): χ2 (d.f. 4) = 21.90, P < 0.001, AIC = 102.85, AUC = 0.767 [95% CI: 0.634, 0.863]					
Intercept		-5.04 (1.40)	12.93	<0.001	
3.	Neurological and psychological problems [yes]	17.45 (3299)*	0.00	0.996	3.8E7 [0, ---]
5.	Time attending preschool [standardized value]	-0.88 (0.33)	6.92	0.009	0.42 [0.22, 0.80]
9.	Time spent on screens [hour]	0.18 (0.10)	3.70	0.055	1.20 [0.99, 1.45]
11.	Communicative interaction with children [Score]	0.26 (0.14)	3.54	0.060	1.30 [0.99, 1.70]

Model 2. A model with three higher data-fitting variables (n = 128): χ2 (d.f. 3) = 19.04, P < 0.001, AIC = 103.55, AUC = 0.762 [95% CI: 0.629, 0.857]

Intercept		-4.97 (1.39)	12.84	<0.001		
5.	Time attending preschool [standardized value]	-0.93 (0.33)	7.77	0.005	0.39	[0.21, 0.76]
9.	Time spent on screens [hour]	0.16 (0.09)	2.91	0.088	1.18	[0.98, 1.41]
11.	Communicative interaction with children [Score]	0.27 (0.14)	3.79	0.052	1.31	[0.99, 1.71]

Model 3. A model with two higher data-fitting variables (n = 128): χ2 (d.f. 2) = 14.94, P < 0.001, AIC = 105.52, AUC = 0.734 [95% CI: 0.601, 0.835]

Intercept		-2.56 (0.52)	24.34	<0.001		
5.	Time attending preschool [standardized value]	-1.02 (0.34)	9.19	0.002	0.36	[0.19, 0.70]
11.	Communicative interaction with children [Score]	0.15 (0.09)	2.72	0.099	1.16	[0.97, 1.39]

Model 4. A model with one best data-fitting variable (n = 128): χ2 (d.f. 1) = 12.27, P < 0.001, AIC = 106.09, AUC = 0.753 [95% CI: 0.640, 0.840]

Intercept		-1.91 (0.30)	41.15	<0.001		
5.	Time attending preschool [year]	-1.02 (0.33)	9.46	0.002	0.36	[0.19, 0.69]

4-year-old (n = 36)

Model 1. Best fit model (n = 36): χ2 (d.f. 2) = 9.14, P = 0.010, AIC = 40.15, AUC = 0.781 [95% CI: 0.576, 0.903]

Intercept		0.70 (0.89)	0.62	0.432		
1.	Sex [female]	1.58 (0.98)	2.63	0.105	4.87	[0.72, 32.99]
5.	Time attending preschool [year]	-1.94 (0.83)	5.42	0.020	0.14	[0.03, 0.74]

Model 2. A model with one best data-fitting variable (n = 36): χ2 (d.f. 1) = 6.18, P = 0.013, AIC = 40.72, AUC = 0.731 [95% CI: 0.550, 0.858]

Intercept		0.85 (0.84)	1.03	0.311		
5.	Time attending preschool [year]	-1.52 (0.70)	4.71	0.030	0.22	[0.86, 4.58]

5-year-old (n = 45)

Model 1. Best fit model (n = 45): χ2 (d.f. 1) = 0.96, P = 0.327, AIC = 42.23, AUC = 0.600 [95% CI: 0.394, 0.772]

Intercept		-0.52 (1.28)	0.17	0.683		
5.	Time attending preschool [year]	-0.56 (0.62)	0.84	0.359	0.57	[0.17, 1.90]

6-year-old (n = 47)

Model 1. Best fit model (n = 47): χ2 (d.f. 2) = 12.15, P = 0.002, AIC = 21.77, AUC = 0.910 [95% CI: 0.610, 0.985]				
Intercept	0.69 (2.79)	0.06	0.804	
5. Time attending preschool [year]	-2.27 (1.07)	4.48	0.034	0.10 [0.01, 0.84]
9. Time spent on screens [hour]	0.55 (0.30)	3.32	0.068	1.73 [0.96, 3.10]
Model 2. A model with one best data-fitting variable (n = 36): χ2 (d.f. 1) = 7.92, P = 0.005, AIC = 23.71, AUC = 0.834 [95% CI: 0.672, 0.925]				
Intercept	3.26 (2.61)	1.55	0.213	
5. Time attending preschool [year]	-2.12 (1.03)	4.21	0.040	0.12 [0.02, 0.91]
